# The mechanism of RNA methylation writing protein-related prognostic genes in lung adenocarcinoma based on bioinformatics

**DOI:** 10.3389/fgene.2025.1541541

**Published:** 2025-06-02

**Authors:** Sha Yin, Guangyan Luo, Lei Luo

**Affiliations:** ^1^ Good Clinical Practice Center, Guizhou Provincial People’s Hospital, Guiyang, China; ^2^ Department of Gastroenterology, Qianxi People’s Hospital, Bijie, China

**Keywords:** lung adenocarcinoma, RNA methylation writer proteins, biomarker, prognostic risk mode, immune cell infiltration

## Abstract

**Objective:**

RNA methylation modifications play biological roles in tumorigenicity and immune response, mainly mediated by the “writer” enzyme. Lung adenocarcinoma (LUAD) development is closely related to RNA methylation. Here, the prognostic values of the “writer” enzymes and the tumor immunosurveillance in LUAD aim to provide new theoretical references for the research of LUAD.

**Methods:**

Genes associated with RNA methylation writer protein in LUAD were identified using The Cancer Genome Atlas Program (TCGA) data and weighted gene co-expression network analysis (WGCNA). Independent prognostic factors were screened by Cox regression and least absolute shrinkage and selection operator (LASSO) regression analyses. A prognostic risk model and a nomogram were established using these genes. Moreover, Gene Set Enrichment Analysis (GSEA) and CIBERSORTx were used to analyze the immune cell infiltration and enrichment pathways in the low- and high-risk groups, respectively. In addition, genes’ potential functions and regulatory mechanisms were explored through gene-gene interaction (GGI) networks and competing endogenous RNA (ceRNA) networks.

**Results:**

We selected 202 genes associated with RNA methylation writer proteins, from which we identified the three genes (CLEC3B, GRIA1, and ANOS1). A prognostic risk model was constructed based on genes associated with RNA methylation writer proteins and stage, demonstrating reliable predictive performance. GGI analysis revealed GRIA1 as a crucial gene. Enrichment analysis revealed that the high-risk group had upregulated pathways connected to cell division. Additionally, immune infiltration analysis revealed that the significantly higher levels of NK cells, activated mast cells, activated CD4 memory cells, and M0 and M1 macrophages displayed in the high-risk group, while the significantly lower levels of monocytes, dendritic cells, M2 macrophages, and inactive CD4 memory cells were in the low-risk group. Moreover, Spearman correlation analysis demonstrated that the three prognostic genes and risk scores correlated highly with various immune cells.

**Conclusion:**

This study identified three prognostic genes related to RNA methylation writer proteins in LUAD. A reliable prognostic model was constructed. The identified prognostic genes also play significant roles in immune cell infiltration in LUAD. This study provides new theoretical references for subsequent in-depth research on LUAD.

## 1 Introduction

Lung cancer is the leading cause of cancer-related mortality globally, with lung adenocarcinoma (LUAD) being the primary pathological subtype, characterized by high mortality and poor prognosis (Sung et al., 2021; Nicholson et al., 2022). Current treatment strategies offer a dismal survival rate, with fewer than 20% of patients with LUAD surviving beyond 5 years (Sung et al., 2021). Despite advancements in diagnostic techniques and the development of targeted therapies, the effectiveness of treatment remains limited, as drug resistance often undermines therapeutic outcomes (Dong et al., 2019). Therefore, identifying novel biomarkers and therapeutic targets and improving the understanding of molecular mechanisms is crucial for the early diagnosis, prognosis, and treatment of LUAD.

Over nearly a decade of research, it has become evident that the accumulation of both genetic and epigenetic alterations drives cancer progression. While somatic genetic aberrations, such as mutations and copy number variations, are critical in tumorigenesis, epigenetic changes occur more frequently and play a more significant role than somatic mutations (Brzezianska et al., 2013). LUAD, in particular, is characterized by the accumulation of epigenetic alterations in the respiratory epithelium (Dumitrescu, 2012). Among the various types of epigenetic modifications, RNA and DNA methylation modifications are of paramount importance (Jones et al., 2016). With the rapid development of specific antibodies and high-throughput sequencing technologies, over 100 different chemical modifications in cellular RNAs have been identified (Barbieri and Kouzarides, 2020). Increasing evidence suggests dynamic RNA modification pathways are closely linked to key processes such as lung cancer cell proliferation, invasion, metastasis, and other biological behaviors (Diao et al., 2023; Liu et al., 2020; Roundtree et al., 2017). RNA methylation, especially N6-methyladenine (m6A), is a common epigenetic modification that plays a key role in regulating gene expression by affecting RNA stability, splicing, translocation, and translation (Han et al., 2023; Luan et al., 2022).

RNA methylation modifications are dynamically and reversibly regulated by three independent components: “writers,” “erasers,” and “readers.” Writer enzymes such as METTL3 and METTL14 can catalyze RNA methylation modifications by adding methyl groups to specific RNA sites, thereby “writing” methylation information (Zhang et al., 2023). Like members of the YTHDF family, reader enzymes specifically recognize and bind to methylation modification sites, further converting modification information into downstream regulatory pathways that affect RNA metabolism (Lan et al., 2021). Eraser enzymes, such as FTO and ALKBH5, remove the methylation groups and reverse methylation modifications for dynamic regulation (Zhang et al., 2021). In LUAD, aberrant expression of these enzymes may affect the proliferation, migration, and invasion of LUAD tumor cells (Huan et al., 2020; Wang et al., 2021).

Meanwhile, RNA methylation significantly impacts immune cell activity, and its role in LUAD is particularly critical. Taking the common m6A methylation as an example, it regulates immune cell development, differentiation, and function. In the case of T cells, m6A modification affects their activation and differentiation and shapes the strength of the anti-tumor immune response (Qin et al., 2024). The altered polarization state of macrophages after RNA methylation modification affects phagocytosis and the killing of tumor cells (Wu et al., 2022). The ability of dendritic cells to take up, process, and present tumor antigens is also regulated by RNA methylation (Ke et al., 2022).

In addition, researchers have previously explored RNA methylation in LUAD. Wang et al. found that four RNA methylation-modified immune molecule subtypes (RMM-I1, RMM- I2, RMM-I3, and RMM-I4) were presented in LUAD (Wang and Shu, 2024). Yang et al. showed that m6AlncRNAs are a reliable prognostic tool that can aid in therapeutic decision-making in ‘driver-gene-negative’ LUAD (Yang et al., 2023). In addition, Li et al. found that methylation-driven lncRNAs and mRNAs contribute to LUAD survival, and four of the lncRNAs and eight of the mRNAs may be potential biomarkers of LUAD prognosis (Li et al., 2019). However, none of these studies have examined RNA methylation writing to proteins.

Thus, this study investigated the prognostic value of RNA methylation writing proteins in LUAD and developed a prognostic prediction model. The potential regulatory mechanisms and immune cell infiltration were also explored, providing a theoretical foundation for individualized treatment strategies in LUAD. udies, which have examined RNA methylation writing to proteins.

## 2 Materials and methods

### 2.1 Data selection

Clinical features, survival data, and RNA-seq transcriptome information for LUAD were obtained from the UCSC Xena TCGA database and served as the training set. The training set comprised 585 cases, including 526 LUAD tumor samples and 59 adjacent normal lung tissue samples. The GSE50081 dataset (GPL570) from the GEO database was used for validation set 1. There were a total of 181 samples in this dataset. This study selected all samples and their matched survival information for subsequent analysis. GSE72094 (GPL15048) was used as validation set 2, which contains 389 LUAD tumor samples. Additionally, 27 RNA methylation writer genes were selected based on previous studies.

### 2.2 Identification of LUAD-associated genes related to RNA methylation writer proteins

Differentially expressed genes (DEGs) between LUAD and control samples were analyzed using the “Deseq2” R package, with a threshold of |log2FC| > 1 and p-value <0.05. Scores of RNA methylation writer protein-related genes were computed using single-sample gene set enrichment analysis (ssGSEA) (P < 0.05), with high scores serving as phenotypic traits to assess correlations with gene modules. Kaplan-Meier (KM) survival curves were utilized to evaluate the association of RNA methylation writer proteins with patient survival (P < 0.05). A scale-free co-expression network was constructed from the training set using the “WGCNA” R package, based on the 27 RNA methylation writer genes. Specifically, the “hclust” function was first used for hierarchical clustering to check for outliers in the samples, and the outlier samples were removed. Then, the optimal soft threshold was set according to the scale-free fit index (signed R2) and the average connectivity (close to 0). Subsequently, genes were divided into different modules according to the criteria of the hybrid dynamic tree-cutting algorithm. The minimum number of genes in each gene module was set to 100. A correlation analysis was conducted between the gene modules and the RNA methylation writing scores, and the module with the highest correlation was selected as the gene-related module for RNA methylation writing. Finally, the genes within the modules were screened based on the criteria of |gene significance (GS)| > 0.2 and |module membership (MM)| > 0.7. The intersection of DEGs and WGCNA-related RNA methylation writer genes was considered candidate genes.

### 2.3 Enrichment analysis and PPI network construction

The R package “clusterProfiler” (adj.p < 0.05) was used to conduct enrichment analysis of candidate genes for the Kyoto Encyclopedia of Genes and Genomes (KEGG) and Gene Ontology (GO). To explore the protein interactions among the candidate genes, a protein-protein interaction (PPI) network was built through the STRING database (confidence value >0.4).

### 2.4 Developing a nomogram and risk model using biomarkers

Prognostic data and the expression profiles of genes related to RNA methylation writer proteins were merged, and univariate Cox regression analysis from the “survival” R package was applied to examine the relationship between gene expression and prognosis (P < 0.05). Genetic screening was conducted using the proportional hazards (PH) hypothesis test (P > 0.05) based on univariate Cox results. The genes that had passed the univariate Cox regression analysis and the PH test were subjected to the least absolute shrinkage and selection operator (LASSO) Cox regression analysis. LASSO achieved feature selection by shrinking the coefficients to 0, ensuring the sparsity and generalization ability of the model. Ten-fold cross-validation was performed, and genes were selected according to the lambda. min value. Subsequently, the risk model was constructed. First, the risk score was calculated using the weighted regression coefficients and the linear combination of expression levels as follows: Risk score = Σ (ExpmRNAn × βmRNAn). Among them, “Exp” represented the expression level of the corresponding gene, and “β” was the regression coefficient of the corresponding gene.

The patients in the training and validation sets were divided into high-risk and low-risk groups according to the median of the risk scores. A risk curve was constructed based on these scores. KM analysis and the log-rank test assessed survival differences between the high-risk and low-risk groups. To evaluate the prognostic utility of the risk model, time-dependent receiver operating characteristic (ROC) curves and the associated area under the curve (AUC) values were calculated for both the training and validation sets (AUC >0.6). Meanwhile, a heat map of the expression levels of prognostic genes between the high-risk and low-risk groups was drawn to understand the expression differences of prognostic genes between the high-risk and low-risk groups. Univariate Cox regression analysis (Hazard Ratio (HR) ≠ 1, P < 0.05) and PH test (P > 0.05) were performed on risk score, age, gender, and stage. Subsequently, factors that passed the univariate Cox regression analysis and PH test were subjected to multivariate Cox regression analysis (HR≠1, P < 0.05) to identify independent prognostic factors. Based on independent prognostic factors, in the training set, the “rms” package was used to construct a nomogram model for predicting the 1-year, 3-year, and 5-year survival rates of LUAD patients. To evaluate the predictive performance of the nomogram model, the calibration curve of the nomogram model was plotted, and decision curve analysis (DCA) was conducted.

### 2.5 GSEA

To reveal the distinctions between the two risk groups’ participation in signaling networks and pertinent biological processes, GSEA was performed. First, the “msigdbr” package in R language was used to download the GO gene sets (c5.go.bp.v2023.2. Hs.symbols) and KEGG gene sets (C2: KEGG gene sets) as the background sets. Subsequently, a differential analysis was conducted between the high-risk and low-risk groups, and the log2FC values were calculated. Then, the genes were ranked in descending order according to the log2FC values. Finally, the GSEA function in R language was used to perform GSEA (adj.P < 0.05).

### 2.6 Analysis of immune cell

Immune cell infiltration in the training set was analyzed using the CIBERSORTx algorithm to assess the role of prognostic biomarkers in the LUAD immune microenvironment. Samples enriched with significant immune cell populations (P < 0.05) were selected based on their immune cell infiltration levels. The Wilcoxon test was applied to compare immune cell enrichment scores between the high-risk and low-risk groups (P < 0.05). A total of 1,000 permutation tests were conducted to evaluate the reliability of the analysis results. To further explore whether the biomarkers and risk scores could predict the infiltration of immune cells during disease progression, Spearman’s correlation analysis was performed using R software to evaluate the relationships between the immune cells with differential immune enrichment scores between the high-risk and low-risk groups and the biomarkers.

### 2.7 GGI analysis and ceRNA network

GeneMANIA (http://genemania.org) was used to construct a gene-gene interaction (GGI) network for the biomarkers to explore the potential functional roles of prognostic biomarkers. Specifically, the gene names were entered into the database, and then the species “*Homo sapiens*” was selected for the construction of the GGI network. The regulatory network of biomarkers was also constructed using a competing endogenous RNA (ceRNA) network, incorporating miRNAs and lncRNAs. miRNAs involved in regulating biomarker expression were predicted using the miRDB and Targetscan databases, while related lncRNAs were identified using the Starbase database. Finally, the Cytoscape software was used to visualize the ceRNA network.

## 3 Results

### 3.1 Identified RNA methylation writer proteins-related genes

**FIGURE 1:**
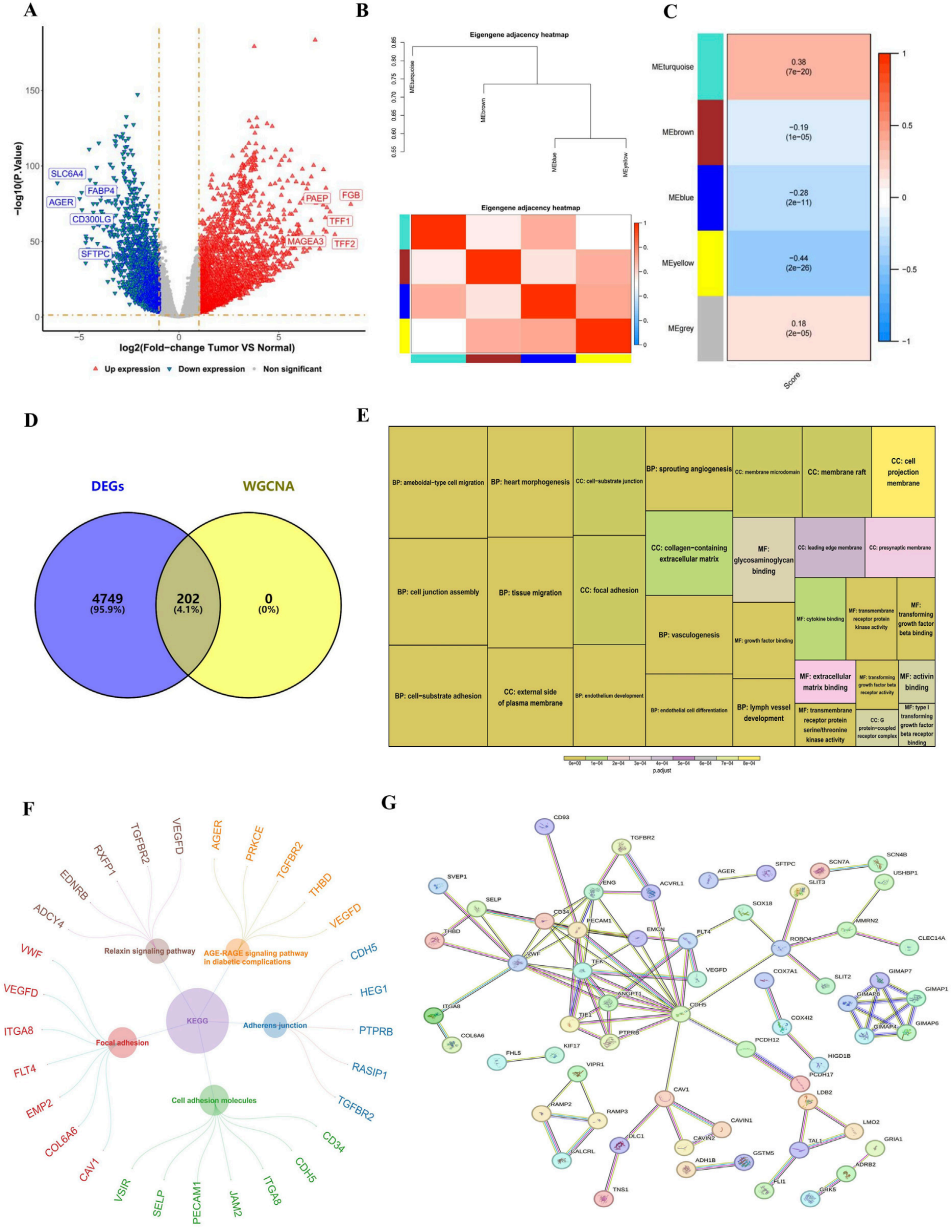
Identified RNA methylation writer proteins-related genes. **(A)** Volcano plot of DEGs. Each dot represents a gene, red and blue dots represent upregulated and downregulated genes, respectively. There were a total of 4,951 DEGs, among which 3,156 were upregulated and 1795 were downregulated. **(B,C)** Heatmap of the four gene modules most correlated with RNA methylation writer proteins. **(D)** Venn diagram showing the 202 candidate genes related to RNA methylation writer proteins. **(E)** GO analysis of candidate genes. The size of the box indicates the number of genes included, and the color indicates significance. **(F)** KEGG analysis of candidate genes. The circle represents the enriched KEGG pathway, and the outer circle is the gene enriched in that pathway. **(G)** PPI network revealing 61 gene nodes. DEGs, differentially expressed genes; GO, Gene Ontology; KEGG, Kyoto Encyclopedia of Genes and Genomes; PPI, Protein-Protein Interaction.

Several analyses were conducted to identify genes associated with RNA methylation writer proteins. Initially, DEGs between the LUAD and normal groups were extracted from the training set, revealing 4,951 DEGs, with 3,156 upregulated and 1795 downregulated (Figure 1A). The ssGSEA scores were calculated based on 27 methylation writer genes, demonstrating a significantly higher level of RNA methylation writing in LUAD tissue compared to normal tissue (Supplementary Figure S1A). Survival analysis via the Kaplan-Meier curve revealed that patients with elevated RNA methylation writing had poorer survival outcomes than those with lower levels (Supplementary Figure S1B). WGCNA, using these scores as phenotypic traits, identified four significant modules, with the most strongly correlated module comprising 202 genes associated with RNA methylation writer proteins (Figures 1B, C). The intersection of DEGs and WGCNA led to identifying 202 candidate genes (Figure 1D).

GO and KEGG enrichment analyses explored the functional roles and pathways of these candidate genes in LUAD progression. GO analysis revealed significant enrichment in biological processes (BP), such as vasculogenesis, heart morphogenesis, and endothelium development; cellular components (CC), including the external side of the plasma membrane and focal adhesion; and molecular functions (MF), such as transmembrane receptor protein kinase activity (Figure 1E). KEGG analysis indicated involvement in pathways related to cell adhesion molecules and adherens junctions (Figure 1F). The PPI network, constructed using the STRING database, identified 61 gene nodes, suggesting that these genes play a pivotal role in LUAD (Figure 1G).

### 3.2 Establishment and validation of prognostic risk model

**FIGURE 2:**
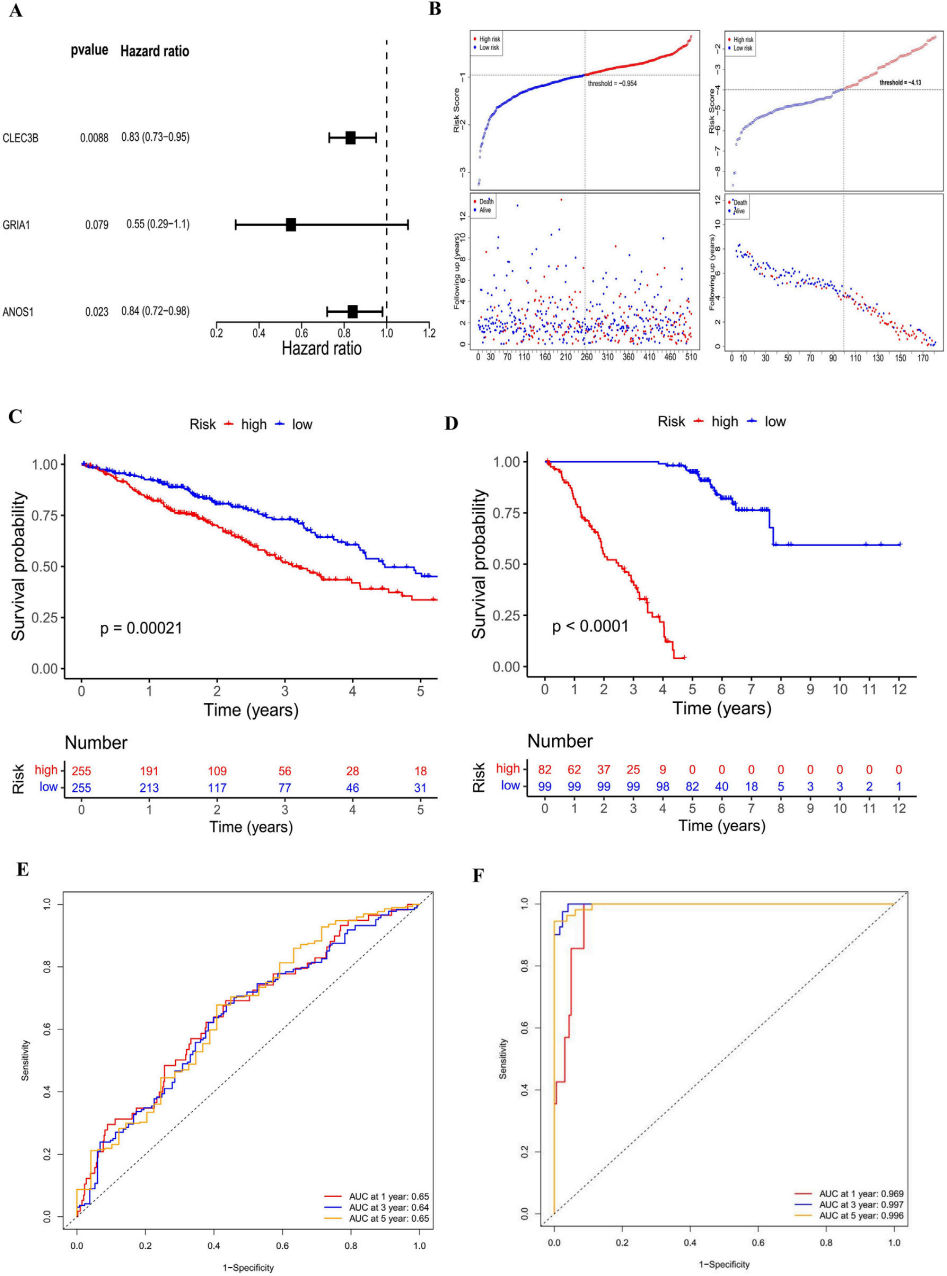
Establishment and validation of the prognostic risk model. **(A)** Forest plots displaying prognostic genes identified through univariate and multivariate Cox regression. Three prognostic genes, CLEC3B, GRIA1, and ANOS1, were identified. They were all protective factors for LUAD. The significance level is set at P < 0.05. **(B)** Risk scores and survival time distribution for patients with LUAD in the training and validation sets. The mortality rate of patients in the high-risk group was higher than that in the low-risk group. A circle represents a sample. **(C, D)** Kaplan-Meier survival curves for high- (training set: n = 255; validation set: n = 82) and low-risk (training set: n = 255; validation set: n = 99) groups in training and validation sets. The survival differences between the high-risk and low-risk groups were compared using the Log-rank test. The survival probabilities of the high-risk group were significantly lower than those of the low-risk group in both the training set (P = 0.00021) and the validation set (P < 0.0001). **(E, F)** ROC curve AUCs for 1-, 3-, and 5-year survival. LUAD, lung adenocarcinoma; AUC, Area Under Curve.

Prognostic genes were selected from the 202 candidate genes using univariate Cox regression analysis, resulting in 118 genes. LASSO regression further refined the list to 9 characteristic genes. Multivariate Cox regression, based on the Akaike Information Criterion (AIC), identified three prognostic genes (they were all risk factors for LUAD)—CLEC3B (HR = 0.83), GRIA1 (HR = 0.55), and ANOS1 (HR = 0.84)—which were subsequently used to construct a prognostic risk model (Figure 2A). The risk score formula is as follows: Risk score = CLEC3B × (−0.183) + GRIA1 × (−0.593) + ANOS1 × (−0.175). Patients were stratified into high-risk and low-risk groups according to the median risk score (training set: −0.954; validation set 1: −4.13; validation set 2: −5.87). The analysis revealed that the high-risk group exhibited significantly higher mortality rates (Figures 2B, C; Supplementary Figure S2A) and lower survival rates in both training and validation cohorts (P < 0.05) (Figures 2D, E, Supplementary Figure S2B). The model’s prognostic accuracy for LUAD was confirmed by ROC curve analysis, with AUC values of approximately 0.65 for 1-, 3-, and 5-year survival in the training set, near 1 in the validation set 1, and approximately 0.67 in the validation set 2 (Figures 2F, G; Supplementary Figure S2C). The AUC values of the ROC curves for 1-, 3-, and 5-year in both the training set and the validation set were all greater than 0.6, indicating that the prediction effect of the risk model was good. Furthermore, CLEC3B, GRIA1, and ANOS1 all had higher expression levels in the low-risk group (Supplementary Figure S3).

### 3.3 Prognostic risk assessment of LUAD patients

**FIGURE 3:**
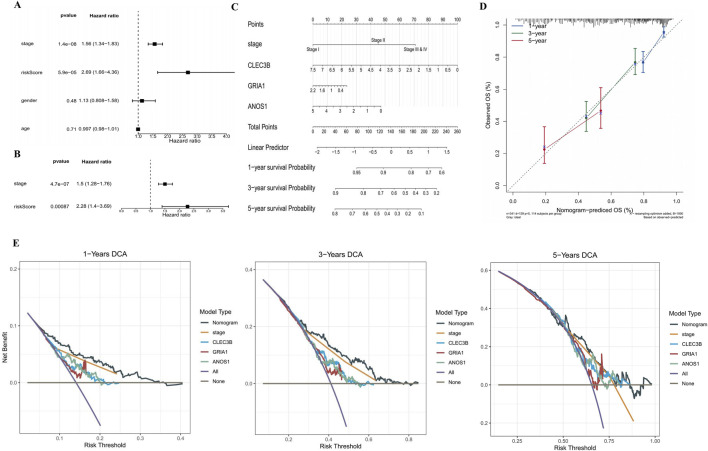
Prognostic risk assessment for patients with LUAD. **(A,B)** Forest plots illustrating HR from univariate and multivariate Cox analyses. Risk score and stage were demonstrated to be an independent prognostic factor. An HR more significant than one is considered a risk factor, while an HR less than one is considered a protective factor. The significance level is set at P < 0.05. **(C)** Nomogram for predicting 1-, 3-, and 5-year survival probabilities of patients with LUAD based on risk score and tumor stage. Each factor corresponds to a point, and the sum of the points of each factor corresponds to the total point. The higher the total point, the lower the patient’s survival rate. **(D)** Calibration curves for 1-, 3-, and 5-year survival predictions based on the nomogram. The slopes of the calibration curves were all close to 1. **(E)** Decision curve analysis for 1-, 3-, and 5-year survival. The curve corresponding to the nomogram exceeded the “All” and “None” baselines, and the net benefit of the nomogram was higher than that of a single independent prognostic factor. HR, hazard ratio.

A nomogram incorporating risk scores and clinicopathological features was developed to predict the risk and survival of patients with LUAD. Both risk score and tumor stage were found to be independent prognostic factors in univariate and multivariate Cox regression analyses and were included in the nomogram (Figures 3A, B). This nomogram estimates the 1-, 3-, and 5-year survival probabilities for patients with LUAD based on risk score and tumor stage (Figure 3C). Calibration curves indicated that the observed overall survival (OS) closely matched the predicted OS for 1-, 3-, and 5-year intervals, demonstrating the nomogram’s accuracy (Figure 3D). Decision curve analysis (DCA) revealed that the nomogram provided more significant net benefits compared to individual factors, further validating its predictive accuracy (Figure 3E).

### 3.4 Enrichment pathways for different risk groups

**FIGURE 4:**
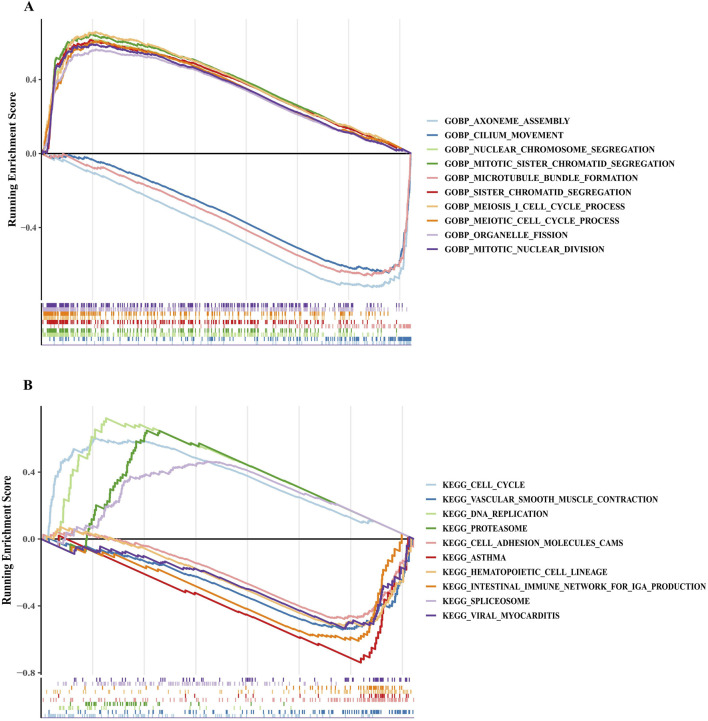
GSEA revealed key pathways in the high-risk and low-risk groups. **(A,B)** Functional enrichment pathways for different risk groups based on GO and KEGG gene sets. A negative enrichment score indicates the downregulation of the pathway, while a positive enrichment score represents the upregulation of the pathway. GO, Gene Ontology; KEGG, Kyoto Encyclopedia of Genes and Genomes.

GSEA was performed to identify functional variations between risk groups. In the high-risk group, GO enrichment analysis revealed the upregulation of functions related to chromosome segregation, mitosis, and meiosis (Figure 4A). Conversely, the low-risk group showed enhanced functions associated with axoneme assembly, cilium movement, and microtubule bundle formation (Figure 4A). KEGG enrichment analysis further indicated that the high-risk group was enriched in pathways associated with the spliceosome, DNA replication, proteasome, and the cell cycle (Figure 4B). In contrast, the low-risk group exhibited enriched pathways related to hematopoietic cell lineage, vascular smooth muscle contraction, cell adhesion molecules, and viral myocarditis (Figure 4B). These results suggest that upregulated cell division-related pathways characterize the high-risk group.

### 3.5 Immune cell infiltration in different risk groups

**FIGURE 5:**
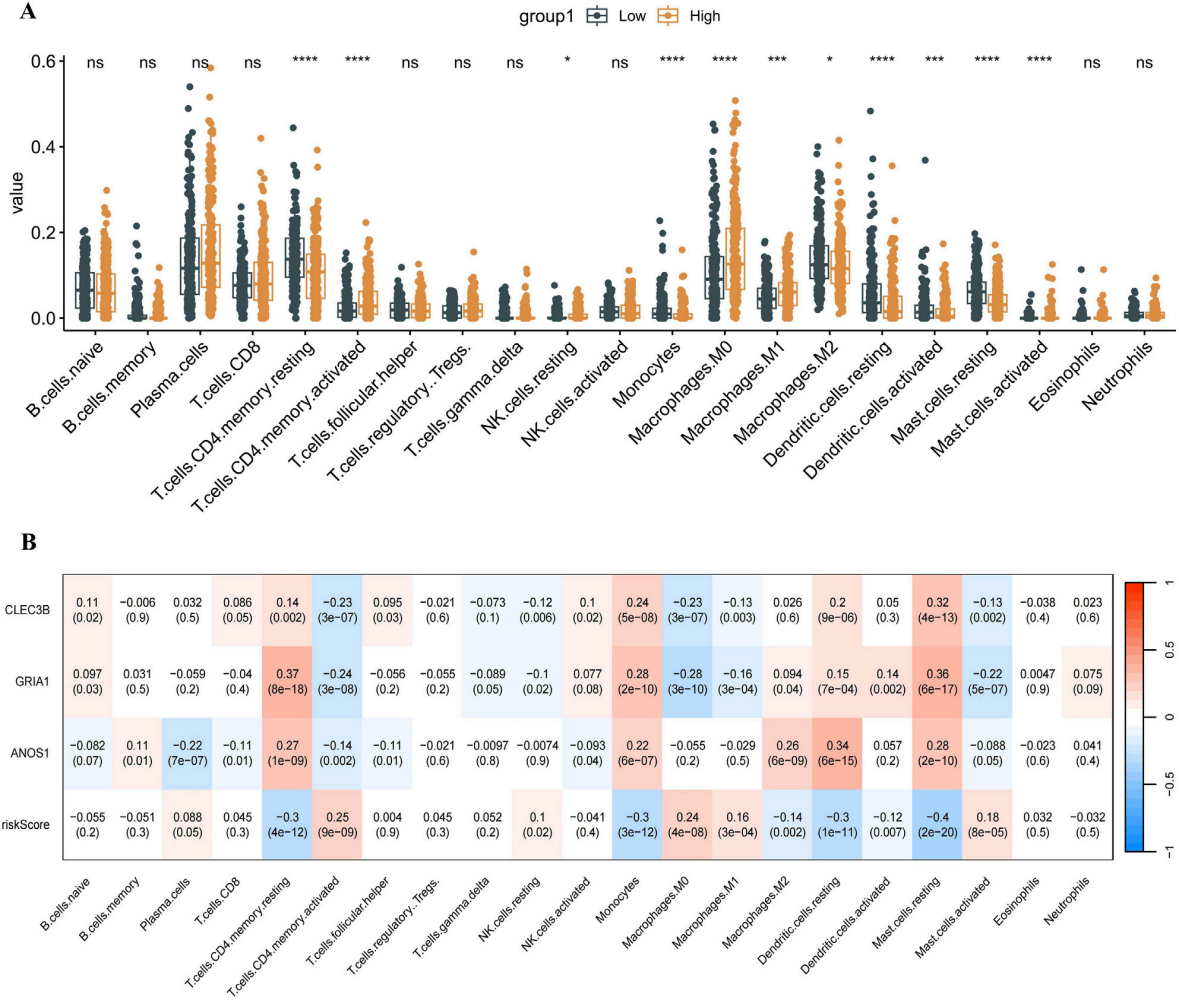
Immune cell infiltration in different risk groups. **(A)** Immune cell infiltration of 21 immune cell types in patients with LUAD is categorized into low-risk and high-risk groups. The Wilcoxon test was used to compare the differences in immune cell infiltration between the high-risk and low-risk groups. 11 types of immune cells showed differences between the two groups (P < 0.05). **(B)** Heatmap showing correlations between prognostic genes, immune cell enrichment scores, and risk scores based on Spearman correlation analysis. The redder the color, the stronger the positive correlation; the bluer the color, the stronger the negative correlation. *P < 0.05, **P < 0.01, ***P < 0.001, ****P < 0.0001, ns. LUAD, lung adenocarcinoma; ns, no significance.

To elucidate the role of prognostic genes in the LUAD immune microenvironment, immune cell infiltration was analyzed using the CIBERSORTx method, which estimates the abundance of immune cell types based on gene expression data. The high-risk group showed increased infiltration of NK cells, activated mast cells, activated CD4 memory cells, and M0 and M1 macrophages. In contrast, monocytes, dendritic cells, M2 macrophages, and inactive CD4 memory cells were reduced (Figure 5A). These results suggest that the inflammatory response at the tumor site may be more intense in the high-risk group. To determine whether the risk model and prognostic genes could reliably predict immune cell infiltration in LUAD, the correlation between prognostic genes, immune cell enrichment scores, and risk scores was examined using Spearman correlation analysis. The results revealed strong correlations between the three prognostic genes, risk scores, and various immune cell types, highlighting their potential as prognostic biomarkers (Figure 5B).

### 3.6 Potential functions and regulatory networks of biomarkers

**FIGURE 6:**
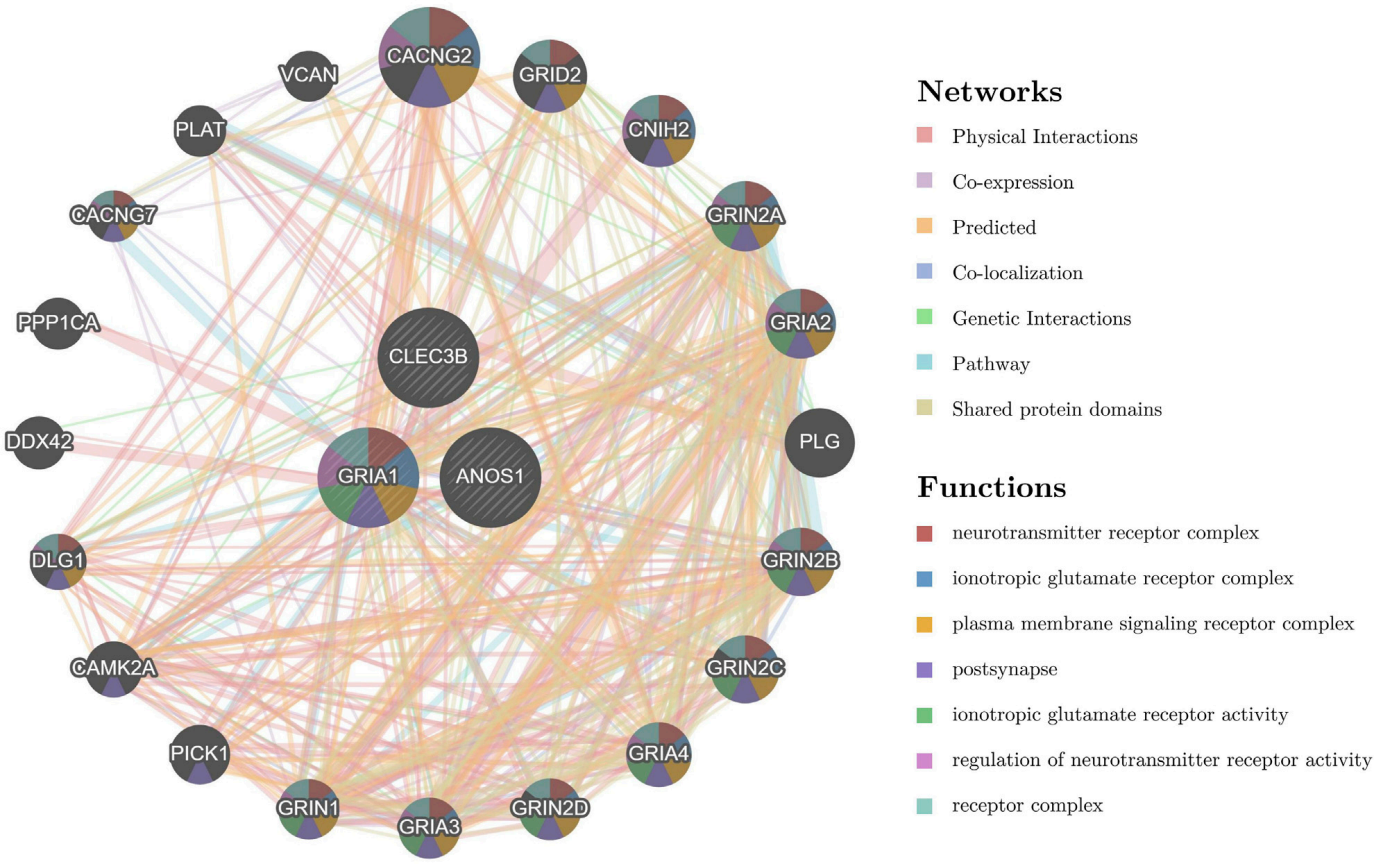
Potential functions and regulatory networks of prognostic genes. The GGI network highlights the top 20 genes (such as CACNG2, GRID2, and CNIH2) and the related pathways of prognostic genes (such as neurotransmitter receptor complex, ionotropic glutamate receptor complex, and plasma membrane signaling receptor complex). The center circle represents the prognostic gene, and the surrounding circle represents the gene associated with the prognostic gene. The color in each circle represents the signaling pathway associated with the gene. The line between the circles represents the interaction between the two genes, and the color of the line represents the interaction pattern between the two genes. GGI, gene-gene interaction.

**FIGURE 7:**
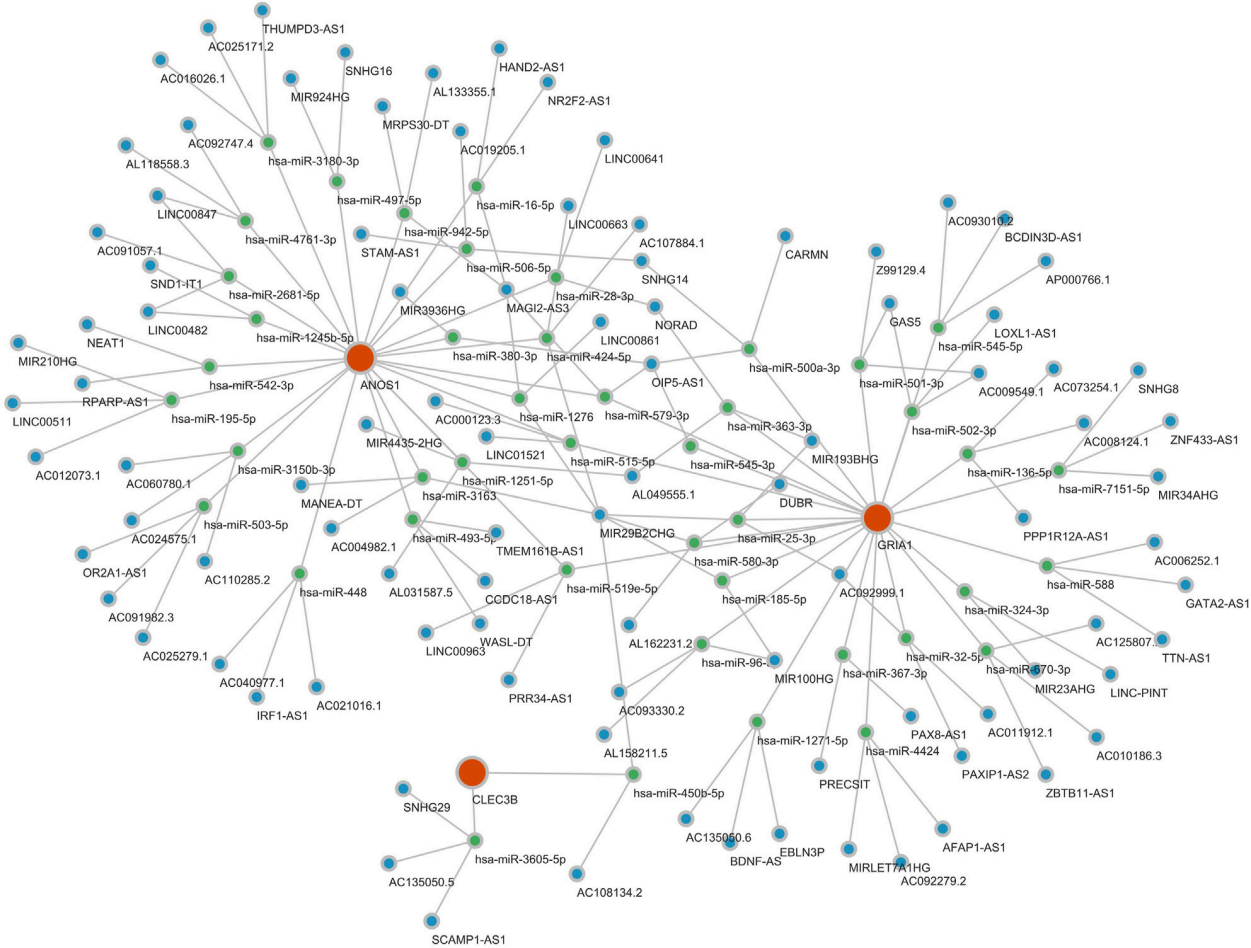
Potential expression regulation mode of prognostic genes. The ceRNA regulatory network analysis identified numerous potential regulatory mechanisms involving 22 miRNAs for GRIA1, 6 for CLEC3B, and 23 for ANOS1, interacting with 463 associated lncRNAs. Red is a gene, green is miRNA, and blue is lncRNA.

A GGI network was constructed to investigate prognostic genes’ potential roles, emphasizing the top 20 genes (such as CACNG2, GRID2, and CNIH2) and their associated pathways. GRIA1 was found to participate in multiple biological functions, including ionotropic glutamate receptor activity, regulation of neurotransmitter receptor activity, and involvement in various receptor complexes, underscoring its critical biological role (Figure 6). A ceRNA regulatory network was analyzed to explore further the regulatory mechanisms influencing biomarker expression. In this network, GRIA1 was involved with 22 miRNAs, CLEC3B with 6 miRNAs, and ANOS1 with 23 miRNAs. There were a total of 463 related lncRNAs. These miRNAs, lncRNAs, and the three prognostic genes constituted a complex ceRNA regulatory network. Complex regulatory relationships such as NEAT1-hsa-miR-542-3p-ANOS1, CARMN-hsa-miR-500a-3p-GRIA1, and SNHG29-hsa-miR-3605-5p-CLEC3B were present therein (Figure 7). These results suggest extensive regulatory networks governing the expression of these prognostic genes.

## 4 Discussion

RNA methylation modifications are closely linked to cancer development and progression, playing a pivotal role in shaping immune cell infiltration within the tumor microenvironment (TME) (Wang and Shu, 2024). However, the molecular mechanisms underlying RNA methylation writer proteins and their diagnostic significance in lung cancer, especially LUAD, remain underexplored.

In this study, RNA methylation writer protein-associated genes related to LUAD prognosis were identified, including CLEC3B, ANOS1, and GRIA1. A novel risk model based on these identified genes was developed, demonstrating its effectiveness in prognostic prediction for patients with LUAD. Additionally, immune infiltration analysis revealed significantly higher levels of natural killer (NK) cells, activated mast cells, activated CD4 memory cells, and M0 and M1 macrophages in the high-risk group compared to the low-risk group. Conversely, the high-risk group showed decreased levels of monocytes, dendritic cells, M2 macrophages, and inactive CD4 memory cells. Spearman correlation analysis further confirmed strong associations between the three prognostic genes and risk scores with various immune cell types.

The GRIA1, CLEC3B, and ANOS1 prognostic signatures demonstrated significant predictive capabilities for patient outcomes in training and validation cohorts. GRIA1, encoding the glutamate ionotropic receptor AMPA type subunit 1, has been identified as a prognostic factor in LUAD (Qian et al., 2022; Wang et al., 2023). Meanwhile, Xu et al. found that the GRIA1 gene was related to the sensory perception of chemical stimulus and the sensory perception of the smell signaling pathway, suggesting that the GRIA1 gene may affect the process of LUAD through these signaling pathways (Xu et al., 2022). Glutamate acts as both a neurotransmitter and a growth factor, promoting the proliferation of normal and malignant cells (Tilley et al., 2017). Dysregulation of glutamate signaling, often due to changes in glutamate receptor expression, has been implicated in various cancers. Additionally, genes linked to glutamate receptor signaling have been found to undergo abnormal methylation in several malignancies (Stepulak et al., 2014; Luksch et al., 2011; Wu et al., 2010). CLEC3B is a transmembrane Ca2+-binding protein in the plasma membrane, extracellular matrix, and exosomes (Dai et al., 2019). The p53 signaling pathway regulates CLEC3B and indirectly modulates the epithelial-mesenchymal transition pathway, influencing LUAD cell invasion and migration. CLEC3B expression is significantly reduced in LUAD, with this downregulation correlating strongly with clinical staging (Lu et al., 2022). In addition, CLEC3B has been studied in other tumors. For example, in cholangiocarcinoma, CLEC3B inhibited cellular proliferation and migration of cholangiocarcinoma through the Wnt/β-catenin pathway (Wu et al., 2024). In clear cell renal cell carcinoma, CLEC3B expression is downregulated and inhibits cell proliferationthe (Liu et al., 2018). ANOS1 is a secreted glycoprotein (100 kDa) constituting an extracellular matrix component (Murcia-Belmonte et al., 2010). ANOS1 plays a role in cell adhesion, neurogenesis, and the motility and migration of neural cells during development (Murcia-Belmonte et al., 2016). Moreover, circulating ANOS1 has been identified as a diagnostic biomarker in gastric cancer (Kanda et al., 2020). The study has shown that ANOS1 is associated with the immune microenvironment of LUAD and is a good predictor of overall survival in LUAD patients (Davoodi-Moghaddam et al., 2024). Moreover, ANOS1 expression was significantly enhanced in esophageal cancer patients and cell lines, and its increased expression was associated with advanced T stage and worse disease-free survival of esophageal cancer patients (Zhu et al., 2024). Our findings align with previous research. GO and KEGG analyses revealed that these RNA methylation-associated prognostic genes are enriched in cellular components like cell adhesion molecules and adherens junctions, which may influence LUAD cell migration.

GSEA further revealed that the high-risk group was predominantly enriched in biological processes related to cell division and the cell cycle pathway. This suggests that the poor prognosis observed in high-risk patients with LUAD may be partly attributed to dysregulation of the cell cycle, which is closely linked to tumor proliferation and progression.

Recent advancements in tumor immunotherapy underscore the critical role of the immune system in lung cancer onset and progression (Camidge et al., 2019; Carbone et al., 2015). Research has established that immune cell infiltration and the tumor microenvironment influence tumor prognosis (Zhang and Zhang, 2020; Muppa et al., 2019; Zhang et al., 2019). Our analysis of immune cell infiltration demonstrated an increased presence of NK cells, activated mast cells, activated CD4 memory cells, and M0 and M1 macrophages in the high-risk group. In contrast, levels of monocytes, dendritic cells, M2 macrophages, and inactive CD4 memory cells were reduced. As a key member of intrinsic immunity, NK cells can rapidly recognize and kill tumor cells, directly lysing lung adenocarcinoma cells by releasing cytotoxic substances, such as perforin and granzyme. They also secrete cytokines, such as IFN-γ, to regulate the tumor microenvironment and inhibit tumor angiogenesis, thus limiting tumor growth and metastasis (Russick et al., 2024; Gillard-Bocquet et al., 2013; Huang et al., 2022; Chockley et al., 2018). Macrophages exhibit heterogeneity in the lung adenocarcinoma tumor microenvironment. Classically activated M1-type macrophages have antitumor activity, phagocytose tumor cells, secrete proinflammatory cytokines and chemokines, recruit immune cells to the tumor site, and enhance immune surveillance (Zhang et al., 2025). However, in the tumor microenvironment, macrophages are often polarized to the M2 type, which secrete cytokines such as IL-10 and TGF-β that promote tumor cell proliferation, angiogenesis, and immune escape, which are detrimental to tumor control (Zhang et al., 2025; Zhou et al., 2024). At the same time, the interaction between NK cells and macrophages also affects the tumor microenvironment. NK cells can regulate macrophage polarization by secreting cytokines, which leads to their conversion to the anti-tumor M1 type; macrophages can affect NK cell activity and function by releasing cytokines (Wang et al., 2019; Cekic et al., 2014). In addition, RNA methylation can also have an impact on immune cells, which in turn affects tumor progression. For example, m^6^A sequencing revealed that m^6^A modifications of multiple genes are altered in m^6^A methyltransferase METTL3-deficient macrophages, affecting macrophage functions (Gu et al., 2023). METTL3-mediated m^6^A RNA methylation promotes anti-tumor immunity in natural killer cells. METTL3 expression was reduced in tumor-infiltrating NK cells, and METTL3 protein expression levels were positively correlated with NK cell effector molecules (Tang et al., 2024). Additionally, higher infiltration of M0 macrophages has been linked to poor prognosis in LUAD (Liu et al., 2017), while increased infiltration of activated mast cells and CD4 memory T cells correlates with a worse prognosis in patients with LUAD (Deng et al., 2022). Spearman correlation analysis revealed a strong association between RNA methylation prognostic genes and risk scores across various immune cell types, indicating a potential link between these genes and immune cell infiltration in tumor tissue. However, further investigation using additional clinical samples and prospective experiments is necessary to elucidate the mechanisms by which RNA methylation prognostic genes impact immunotherapy responses in LUAD.

In this study, we analyzed public databases and determined that CLEC3B, ANOS1, and GRIA1 are associated with the prognosis of LUAD. However, there are some limitations in this study, such as the biological mechanisms and functions of CLEC3B, ANOS1, and GRIA1 remain unclear. Moreover, the specific mechanism by which RNA methylation regulates the activity of these genes is still unclear. Therefore, in the future, we will collect clinical samples to detect the expression and RNA methylation levels of target genes and analyze their specific associations with the prognosis of LUAD. Secondly, we will evaluate their effects on cancer cell proliferation, migration, and invasion through knockout/overexpression models and explore the potential efficacy of targeted therapies using animal models. In addition, its biological role was revealed through cell function experiments (proliferation, migration) and *in vivo* experimental systems to provide a basis for clinical translation.

## 5 Conclusion

In this study, we successfully identified three prognostic genes associated with RNA methylation writing proteins in LUAD and constructed a reliable prognostic model. These prognostic genes have also been shown to significantly influence immune cell infiltration in LUAD. These findings provide new insights into the regulatory mechanisms of LUAD and provide a reliable theoretical basis for subsequent in-depth studies.

According to the expression level of specific prognostic genes in patients, the risk of disease progression can be predicted, and high-risk patients can be intensified therapeutic intervention in advance. The differential immune cell status reflects the patient’s immune microenvironment characteristics. For patients with low immune cell activity, immune activation therapy can be used to enhance the tumor-killing ability of immune cells; for the abnormal enrichment of specific immune cells, the relevant pathways can be targeted for inhibition to correct the imbalanced microenvironment to achieve precise and personalized lung adenocarcinoma treatment and to improve therapeutic efficacy and the quality of patient’s survival. Meanwhile, more precise treatment strategies can also be formulated according to the RNA methylation status of patients, especially for patients with abnormal RNA methylation modifications. At the same time, RNA methylation inhibitors may enhance the therapeutic response of immune checkpoint inhibitors (e.g., PD-1/PD-L1 inhibitors) in patients, thus providing a strategy for combination therapy.

## Data Availability

The original contributions presented in the study are included in the article/Supplementary Material, further inquiries can be directed to the corresponding author.
