# Supplementary material for: The mechanism of RNA methylation writing protein-related prognostic genes in lung adenocarcinoma based on bioinformatics
Source: Front Genet. 2025 Jun 2;16:1541541. doi: 10.3389/fgene.2025.1541541 (PMC12171300; doi:10.3389/fgene.2025.1541541)
Supplement: Supplementary file 1 [file DataSheet1.pdf]

Supplementary Material

1. Supplementary Figures

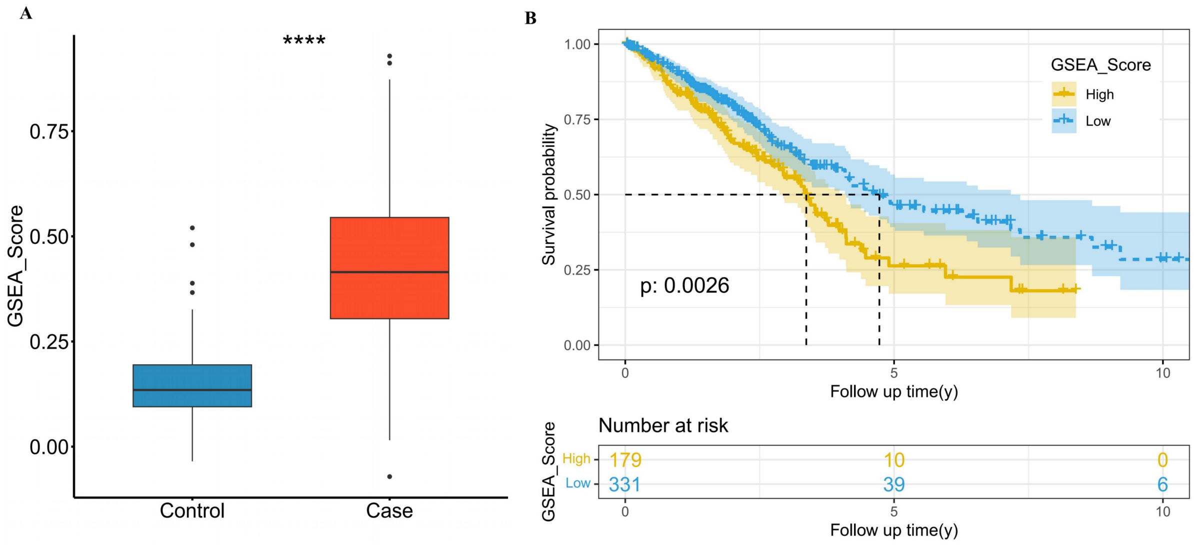

Figure S1. (A) GSEA scores were calculated based on 27 methylation writer genes in LUAD tissue compared to normal tissue. (B) Kaplan-Meier curves of survival probability between high and low GSEA scores, ****P < 0.0001.

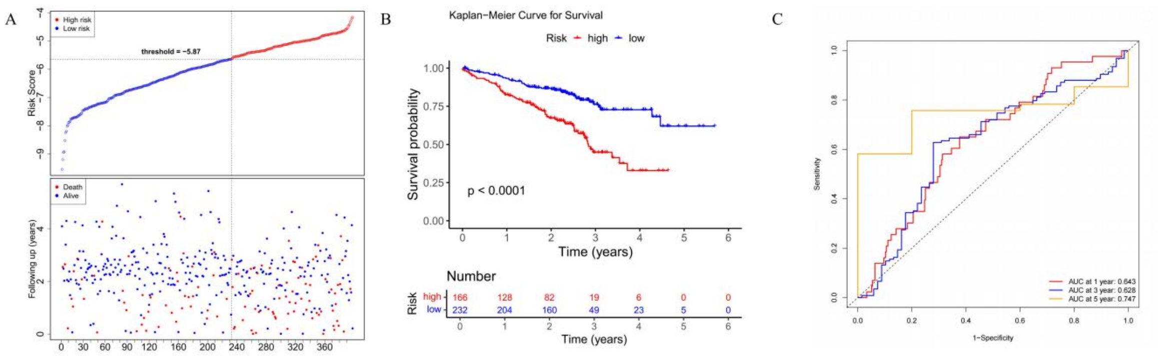

Supplementary Figure 2. Validation of the risk model. (A) Distribution of risk score and survival time of patients with LUAD in validation set 2. The mortality rate of patients in the high-risk group was higher than that in the low-risk group. A circle represents a sample. (B) Kaplan-Meier curves of overall survival time between high- and low-risk groups in validation set 2. The survival differences between the high-risk and low-risk groups were compared using the Log-rank test. The survival probabilities of the high-risk group were significantly lower than those of the low-risk group in validation set 2 ($P < 0.0001$). (C) The areas under the AUCs at 1, 3, and 5-year time points. LUAD, lung adenocarcinoma; AUC, Area Under Curve.

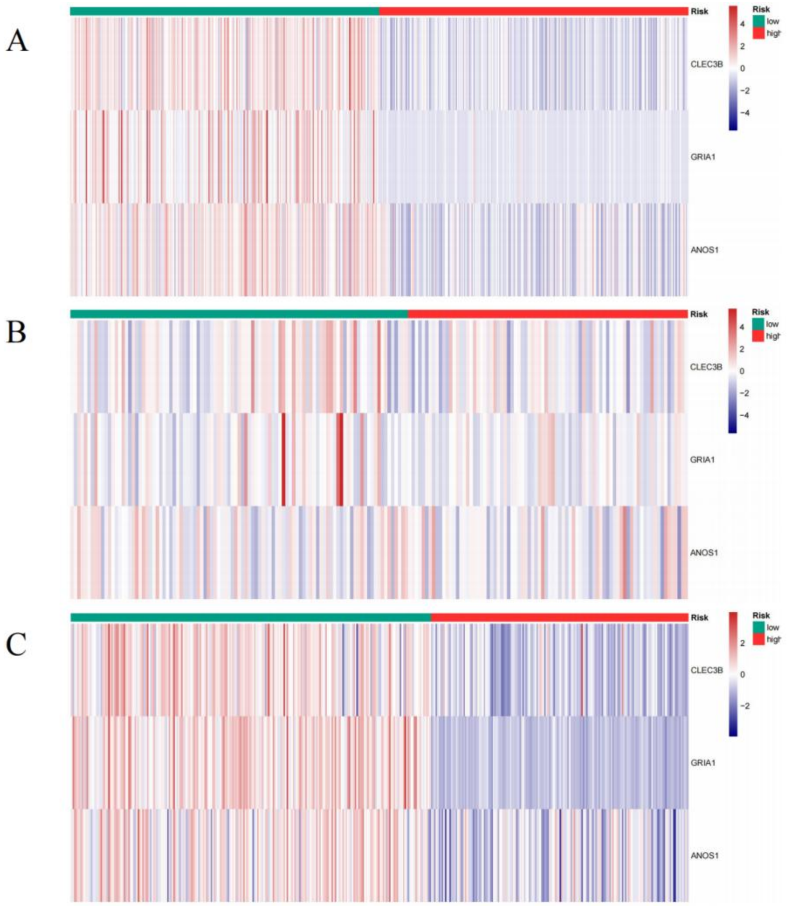

Supplementary Figure 3. Heat map of the expression of CLEC3B, GRIA1, and ANOS1 between the high-risk and low-risk groups. (A) Training set. (B) Validation set 1. (C) Validation set 2. Red indicates higher expression levels, while blue indicates lower expression levels.
